# Brearley’s Systematizer

**Published:** 1882-01-20

**Authors:** 


BREARLEY'S SYSTEMATIZER.—This is a new and convenient diary for keeping memoranda of office work. There is a place and a page for every day in the year, where slips or memoranda of any kind may be deposited, and so arranged as may be quickly and readily found. It will last for many years, and is certainly an unique and happy device for facilitating and systematizing office work. Price, $3.50. Address W. H. Brearley, Detroit, Mich.

